# SARS-CoV-2 secondary attack rates and risks for transmission among agricultural workers and their households in Guatemala, 2022-2023

**DOI:** 10.1016/j.ijregi.2025.100676

**Published:** 2025-05-27

**Authors:** Joseph Daniel Carreon, Molly M. Lamb, Anna N. Chard, Diva M. Calvimontes, Chelsea Iwamoto, Neudy Rojop, Jose Monzon, Ian D. Plumb, Edgar Barrios, Julio del Cid-Villatoro, Kareen Arias, Melissa Gomez, Claudia Maribel Paiz Reyes, Maria Renee Lopez, May Chu, Beatriz Lopez, Bradley S. Barrett, Kejun Guo, Mario Santiago, Guillermo Antonio Bolanos, Emily Zielinski-Gutierrez, Eduardo Azziz-Baumgartner, Eva Leidman, Ashley Fowlkes, Edwin J. Asturias, Celia Cordon-Rosales, Daniel Olson

**Affiliations:** 1 Centers for Disease Control and Prevention (CDC), Atlanta, USA; 2 Department of Epidemiology, Colorado School of Public Health, Aurora, USA; 3 Center for Global Health, Colorado School of Public Health, Aurora, USA; 4 Center for Human Development, Fundación para la Salud Integral de los Guatemaltecos, Retalhuleu, Guatemala; 5 Center for Health Studies, Universidad del Valle de Guatemala, Guatemala City, Guatemala; 6 Division of Infectious Diseases, University of Colorado School of Medicine, Aurora, USA; 7 Department of Pediatrics, University of Colorado School of Medicine, Aurora, USA

**Keywords:** SARS-CoV-2, Household transmission, Secondary attack rates, Guatemala, agricultural worker health, Asymptomatic transmission

## Abstract

**Objectives:**

It is unclear whether agricultural workers working during epidemics frequently introduce respiratory infections into their homes and trigger secondary transmission. We evaluate secondary attack rates (SAR) and transmission risk in households of agricultural workers in Guatemala during the COVID-19 pandemic.

**Methods:**

Households of participants in a workplace surveillance cohort were enrolled from September 2021 to August 2023. All participants reported symptoms twice weekly and provided saliva weekly for SARS-CoV-2 reverse-transcriptase-polymerase chain reaction testing. Upon SARS-CoV-2 detection, participants submitted saliva three times per week for 4 weeks. We calculated SARs, and we estimated the risk of transmission to household contacts adjusting for demographic factors, COVID-19 vaccination status, seropositivity, and significant covariates (p ≤ 0.05) in univariable analyses.

**Results:**

Among 83 households with 376 individuals, 48 (58%) had at least one SARS-CoV-2 infection (120 SARS-CoV-2 infections, 0.6 per 100 person-weeks), resulting in 64 secondary (SAR = 0.35, 95% confidence interval [CI] 0.28-0.43) and eight tertiary infections (tertiary attack rate = 0.07, 95% CI 0.03-0.13). The risk of secondary transmission increased by 112% among household contacts whose index cases were positive for ≥11 days (risk ratio: 2.12, 95% CI 1.29-3.49) but did not increase for those whose index case was positive for 6-10 days (risk ratio: 1.40, 95% CI 0.77-2.57) compared to those with index cases positive for ≤5 days.

**Conclusions:**

More than half of agricultural households became infected with SARS-CoV-2 and approximately two-thirds of these had secondary chains of transmission, especially when index cases shed SARS-CoV-2 longer.

## Highlights

• Overall, 58% of households had ≥1 SARS-CoV-2 infection, with 63% experiencing secondary transmission.
• Most (71%) index cases were adults, with over half working outside the home.
• A total of 27% of transmissions occurred from asymptomatic or pre-symptomatic individuals.
• Longer SARS-CoV-2 RNA shedding was associated with increased risks of secondary infections.
• Agricultural workers should be prioritized for vaccines and non-pharmaceutical interventions.

## Introduction

While agricultural workers may be at increased risk of getting ill during epidemics [1,2], it is unclear if they also typically introduce respiratory infections into homes and trigger secondary and tertiary chains of transmission within their households. Observational data suggest that agricultural workers may be at an increased risk of SARS-CoV-2 infection that may be transmitted within their households [3] increasing disease spread in the community. In turn, agricultural communities affected by infectious respiratory disease may affect commodity production and downstream economies [4].

Agricultural workers play a vital role in the production of global food commodities [5] and comprise a significant proportion of the overall workforce in low- and middle-income countries (LMICs) [6]. Agricultural workers are likely at increased risk for poor clinical outcomes owing to laborious work and a high prevalence of poverty, food insecurity [7], chronic diseases [8], low health care availability, or occupational health resources [9]. During the COVID-19 pandemic, agricultural production continued despite an increased prevalence of SARS-CoV-2 infection among agricultural workers; for example, compared to non-farmworkers, SARS-CoV-2 test positivity was 28% higher among farmworkers living in the same communities in Monterey, California [2]. Additionally, serologic evidence from our study in Guatemala indicated that nearly half of agricultural workers were infected with SARS-CoV-2 in 2020 and nearly three-quarters by 2021 [1] and those with COVID-19 frequently reported difficulty concentrating, irritability, worse clinical and well-being scores, and greater absenteeism and lost income compared to people with other respiratory infections [10].

Although our previous analyses demonstrated high seroprevalence of SARS-CoV-2 in agricultural workers [1] few studies have assessed whether workers frequently introduce infections into their homes or the risk factors for triggering secondary and tertiary transmission chains within households [3,11]. While studies from urban and peri-urban households document how transmission of SARS-CoV-2 varies by variant, the presence of symptoms among index cases, and household isolation measures, it is unclear if these affect transmission in rural households common in agricultural communities in Mesoamerica [11,12]. Transmission in agricultural worker households may be particularly complex due to factors unique to the agricultural sector, such as suboptimal working conditions, and insufficient access to healthcare resources, including vaccinations, which may all contribute further to transmission risk [6,9,13].

Through the AGRIcultural worker COVID-19 Asymptomatic and SymptomAtic transmission in the Home and Workplace (AGRI-CASA) Study, we assessed the frequency with which agricultural workers introduce SARS-CoV-2 into their households and the likelihood of secondary household transmission by clinical, environmental, and household characteristics. We estimated secondary attack rates (SAR), evaluated individual and household characteristics associated with transmission, and assessed episodic data to provide an overview of transmission patterns. This prospective cohort study provided real-time data collection before and during household infections, unlike most retrospective studies, enabling the assessment of asymptomatic and presymptomatic transmission. It provided data on transmission patterns within rural agricultural worker communities, a vulnerable and essential population with limited existing data, and whose findings may apply to other critical agricultural settings in the Americas that have not been extensively studied.

## Methods

### Study setting and population

The AGRI-CASA study was conducted in two rural, resource-limited communities in the coastal lowlands of Southwest Guatemala, approximately 50 km from the border of Mexico. The populations living in these communities suffer from high levels of food insecurity, poverty, low access to healthcare, and high levels of diarrheal, respiratory, and arboviral disease [7,14]. Many of the adults are employed in large agribusinesses, including banana and palm oil plantations, and some participate in a separate agricultural workers and respiratory illness impact (AGRI) study, a prospective cohort of banana plantation workers that carries out active work-based and sentinel surveillance for COVID-19-like Illness with polymerase chain reaction (PCR) testing for SARS-CoV-2.

For the present cohort study, an initial convenience sample of 70 households from the two communities was screened and enrolled. Households that ended participation were replaced to maintain the cohort of at least at 70 households for the duration of the study. Initial enrollment took place from September 2021 to January 2022, and this analysis period continued through August 2023. The enrollment criteria included having more than one member of the household employed at a large agribusiness that participated in the AGRI study, residing within the two study communities, and at least 75% of household members consenting to participate. We defined household members as those who slept under the same roof >50% of the time. Individuals who left the household temporarily were censored until the time of return, and those who did not return were excluded.

### Data collection and routine surveillance

Following informed consent and assent, as appropriate, study nurses collected standardized demographic, clinical, and epidemiologic data, including individual and household-level characteristics, and a venous blood specimen. Household-level characteristics included the number of people in the household, area of the house (m^2^), number of rooms, number of beds, number of windows, monthly household income, number of adults (age ≥18 years) and children (age <18 years) residing in the home. Individual-level characteristics included age group (<5, 5-17, 18-49, 50-64, and ≥65 years), sex, ethnicity, school attendance, work-from-home status, work location (indoor or outdoor), occupation, vaccination status, COVID-19 vaccination dates, medications, and self-reported medical conditions, including kidney disease, anemia, cardiovascular disease, diabetes, liver disease, asthma, obesity (body mass index >30 kg/m^2^), and neurologic disease.

### COVID-19-like illness surveillance

All enrolled participants were screened twice weekly for COVID-like illness (CLI), defined as ≥1 of the following symptoms for >48 hours: fever/chills, cough, shortness of breath, fatigue, muscle/body ache, headache, loss of taste/smell, sore throat, congestion, nausea, vomiting, or diarrhea. Symptom data were summarized from routine surveillance and illness surveys.

### Specimen collection

Each week, regardless of symptom screening results, 5 ml of saliva was collected for virologic testing. In addition to weekly saliva collection, participants with symptoms meeting CLI criteria or with confirmed community or household exposure to SARS-CoV-2 in the past 3 days received an additional visit for saliva collection. Subjects with negative PCR results for SARS-CoV-2, influenza virus, or respiratory syncytial virus (RSV) returned to routine surveillance and were eligible to be re-tested the following week if CLI was reported. For households with an incident SARS-CoV-2 PCR detection, all enrolled household contacts underwent intensive surveillance, providing symptom, epidemiologic data, acute (at week 1) and convalescent blood specimens (at week 6), and saliva three times per week for 4 weeks, regardless of their symptom or laboratory results. Households then returned to routine surveillance. In weeks 5 and 6, participants were administered weekly symptom and long-COVID surveys.

### Specimen testing

All saliva specimens were transported on ice with temperature monitoring to the Center for Human Development, centrally located in the study catchment area. Specimens were refrigerated upon arrival and serum was separated within 24 hours, and then stored at −80°F. All specimens from symptomatic individuals identified during routine surveillance were tested within 24 hours by using, off-label for saliva [15], Roche point-of-care (POC) Cobas® Liat real-time reverse transcription-PCR (RT-PCR) Influenza A/B & RSV and SARS-CoV-2 kits. POC PCR results were shared with participants typically within 24 hours. Specimens from households collected after the incident detection of SARS-CoV-2, influenza virus, or RSV, were tested at a later date by RT-PCR testing (CDC Protocol) [16] at Universidad del Valle de Guatemala or digital droplet PCR at Colorado State University (Fort Collins, CO) [17]. Retrospectively, saliva collected from all household contacts during routine surveillance in the 2 weeks preceding the incident household viral detection was tested in batches later. POC and batched RT-PCR results were shared weekly with the Guatemala Ministry of Health. Since specimens collected in the absence of symptoms were taken after the incident household infection (i.e., “household intensive surveillance”) and were batch-tested, participants could not be informed of their case status in real time.

Sera was shipped to the University of Colorado (Aurora, CO) and tested for anti-nucleocapsid (N) and anti-spike total immunoglobulin (Ig) G using the Roche Diagnostics Elecsys® anti-SARS-CoV-2 immunoassay, as previously described [1]. All specimens were run in triplicate on the Roche Elecsys® e801 per the manufacturer’s instructions.

### COVID-19 vaccination

COVID-19 vaccination dates, doses, and manufacturer data were collected directly from the Guatemalan Ministry of Public Health’s centralized database. In Guatemala, individuals 12 years old became eligible to receive COVID-19 vaccination in September 2021. Vaccination status by time of household infection was defined by number of doses received: unvaccinated (zero), partially vaccinated (one), fully vaccinated (two), and fully vaccinated + booster (three or more). Individuals with no evidence of vaccination were considered unvaccinated. Vaccination timeliness was defined dichotomously by whether any vaccine dose was received within 6 months before the household infection date.

### Index and secondary case definition

An index or co-index case was defined as the first person(s) who tested positive for SARS-CoV-2 within a household. A secondary case was defined as a household contact who tested positive for SARS-CoV-2 during 1-14 days after the index case date [[18], [19], [20], [21], [22]] tertiary case was defined as any household contact who tested positive for SARS-CoV-2 15-42 days after the index case. Forty-two days after the incident index case infection, the household was considered clear of infection and susceptible to subsequent infection. Number of days positive for SARS-CoV-2 was calculated as days between the first and last day of PCR-positive test results within the 42-day window. To assess asymptomatic transmission we classified index cases as “asymptomatic” at onset if there were no reported symptoms 2 days before or within 5 days after the PCR-positive test result.

### Statistical analysis

For continuous variables, medians, and interquartile ranges (IQR) we calculated household- and individual-level characteristics stratified by infection status. Characteristics of household contacts were evaluated according to infection status. The SAR was calculated as the proportion of household contacts with RT-PCR-confirmed SARS-CoV-2 RNA in their saliva (i.e., infection) within 14 days of index case infection; point estimates and 95% confidence intervals were calculated for each household infection episode and plotted by potential risk factors for the first household episode.

We assessed each individual- and household-level risk factor independently for secondary infection using a univariable general estimating equation (GEE) specified with a Poisson distribution, a cluster ID for household, an independent correlation structure to simplify correlation assumptions, and robust standard errors to mitigate potential misspecification of the correlation structure. From these, we estimated unadjusted risk ratios (RRs) and 95% confidence intervals (CIs). For inclusion in the final multivariable GEE model, we selected covariates that achieved significance (*P* ≤0.05) in univariable models. Irrespective of significance in univariable analyses, all final models were adjusted for age group, sex, baseline SARS-CoV-2 seropositivity, and COVID-19 vaccination timeliness. Only the first household episodes were included in risk factor analyses. Households with more than one index case (co-index cases) were excluded from risk factor and SAR analyses. All statistical analyses were performed using R version 4.4.

### Ethics

The study was approved by the Colorado Multiple Institutional Review Board (COMIRB), under protocol #21-2551 (see 45 C.F.R. Part 46 and 21 C.F.R. Part 56.), the Guatemala Ministry of Health National Ethics Committee (HRMC-225-01-2021), and CDC ethics committees. The local Southwest Trifinio Community Advisory Board for Research agreed to the study. Study participants were compensated between $0.67–3.33 USD per visit dependent on visit type and duration. The study was funded by the CDC (CDCGH002243).

## Results

### Households with SARS-CoV-2 infection

**Figure 1:**
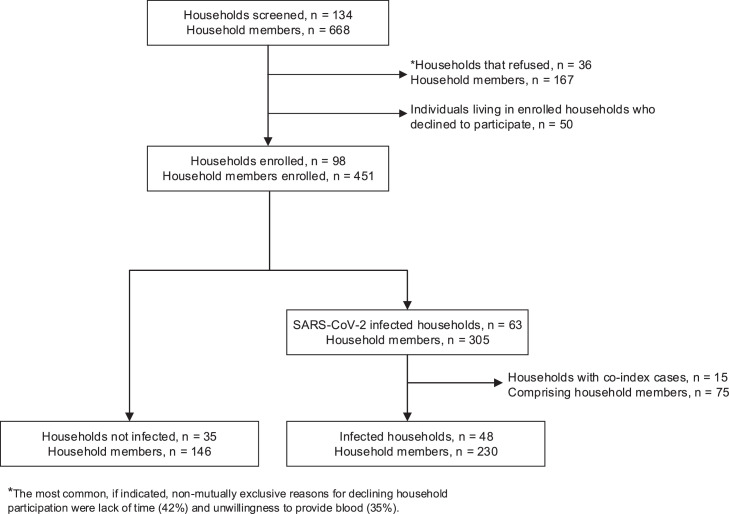
Study households and participants flowchart.

From September 28, 2021, through August 2023, we followed 83 households with 376 individuals (Figure 1) for a median of 210 days (IQR = 65-379). Thirty-five (42%) households had no SARS-CoV-2 infections and 48 (58%) had at least one SARS-CoV-2 infection, 14 of these 48 (29%) with two SARS-CoV-2 reintroductions, and five households (10%) with three reintroductions (Supplementary Figure 1). Overall, the median duration of household infection was 10.5 days (IQR 2–21). Nineteen of 48 (40%) households had only an index SARS-CoV-2 infection, 29 of 48 (60%) had secondary transmission, and 8 (17%) had tertiary transmission. The median time between an index case’s first PCR-positive result and a secondary case was 7 days (IQR 5.8-11) and 11 days (IQR 7.5-14.5) between a secondary and tertiary case. The serial interval was 7 days (IQR 3.8-9.0) between an index case and a secondary case, and 11 days (IQR 7.5-14.5) between a secondary case and a tertiary case.

### Individual risk of SARS-CoV-2 infection

**Figure 2:**
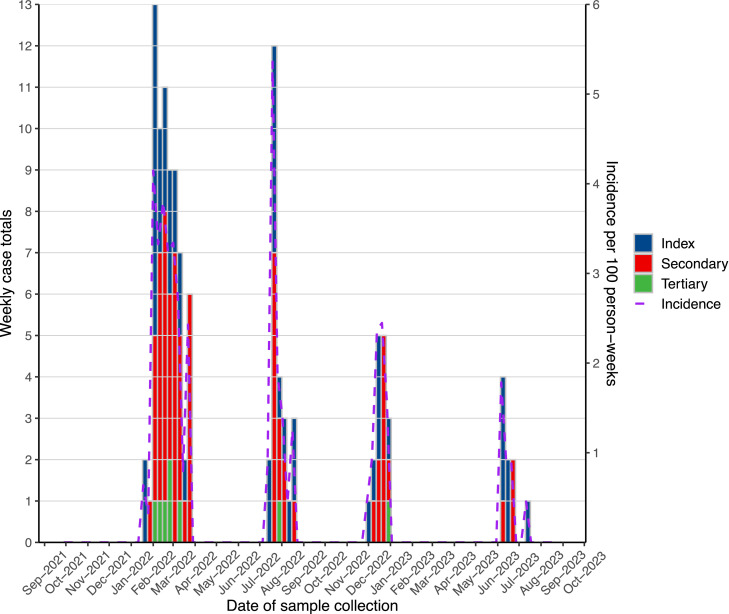
Weekly SARS-CoV-2 infections by case type (n = 120).

The 376 individuals in the 83 study households were 52% female, the majority were aged 5-17 (n = 129, 34%) or 18-49 years (n = 176, 47%), and 89% self-identified as mixed Spanish and Indigenous descent (Table 1). Among 121 household members who identified as working outside of the home, 100 (83%) reported working in the field (n = 78, 64%) or packing plant (n = 22, 18%). Among 266 who were eligible for COVID-19 vaccination, there were 86 (32%) participants that were unvaccinated against COVID-19, 46 (17%) were partially vaccinated, 79 (30%) fully vaccinated, and 55 (21%) fully vaccinated and boosted. More than half of the participants (211, 56%) provided baseline sera, of whom 160 (76%) had anti-N IgG antibodies. Participants who met analysis criteria contributed 20,631 person-weeks and accrued a cumulative 120 infections (0.6 infections per 100 person-weeks) during four SARS-CoV-2 waves (Figure 2); these included 48 (40%) index infections, 64 (53%) secondary infections, and 8 (7%) tertiary infections. Thirteen (27%) index infections were classified as asymptomatic but of whom approximately half (6, 46%) subsequently developed COVID-19 symptoms a median of 8.5 days later (IQR 7-11); one person developed COVID-19 complications and required hospitalization. Additionally, approximately half, 31 of 64, secondary cases (48%) reported symptoms at the time of their sample collection. The percentages of SARS-CoV-2 infection among all individuals did not differ by sex, COVID-19 vaccination, baseline serology, occupation, or work environment. However, 34 out of 48 (71%) index cases were ≥18 years old compared to 94 out of 182 (52%) of non-index cases who were ≥18 years old (χ²(1) = 5.7, *P* = 0.02). More than half of these adult index cases (56%) reported working outside the home.

**Table 1:** Characteristics of households and participants by SARS-CoV-2 infection status.

Household characteristics	All households	Uninfected households	SARS-CoV-2 infection in household		
			No secondary transmission	Secondary or tertiary transmission	
	N = 83^a^	N = 35 (42%)^a^	N = 18 (22%)^a^	N = 30 (36%)^a^^,^^b^	*P*-value^c^
**Number of household members**	5 (4, 6)	4 (3, 5)	5 (4, 5)	5 (4, 6)	0.32
Number of adults (≥18 years)	2 (2, 3)	2 (2, 3)	2 (2, 3)	3 (2, 4)	0.37
Number of children (<18 years)	2 (1, 3)	2 (1, 3)	3 (1, 3)	3 (1, 3)	0.70
**Area of house (m^2^)**	10 (8, 12)	10 (8, 10)	10 (8, 12)	11 (9, 18)	0.37
Square meters per person	2 (1, 4)	2 (2, 3)	2 (1, 4)	2 (1, 5)	0.68
**Number of rooms**	4 (3, 5)	4 (3, 5)	4 (3, 5)	4 (4, 6)	0.41
Rooms per person	0.80 (0.60, 1.20)	0.80 (0.60, 1.25)	0.78 (0.60, 1.20)	0.80 (0.60, 1.17)	0.97
**Number of beds**	3 (2, 4)	3 (2, 4)	3 (3, 4)	4 (3, 6)	0.60
Beds per person	0.70 (0.50, 1.00)	0.67 (0.50, 1.00)	0.78 (0.60, 1.00)	0.73 (0.60, 1.00)	0.60
**Number of windows**	2 (1, 4)	2 (1, 3)	2 (0, 3)	4 (2, 5)	0.44
Windows per person	0.50 (0.25, 0.83)	0.40 (0.25, 1.00)	0.45 (0.00, 0.86)	0.50 (0.33, 0.83)	0.81
**Monthly income (US $)**	387 (323, 452)	348 (310, 413)	374 (323, 426)	413 (323, 452)	0.48


**Individual characteristics**	All participants	No household infections	Exposed but uninfected	Infected	
	N = 376^a^	N = 146 (39%)^a^	N = 110 (29%)^a^	N = 120 (32%)^a^	
**Age group**					0.07
<5	36 (9.6%)	18 (12%)	12 (11%)	6 (5.0%)	
5-17	129 (34%)	45 (31%)	44 (40%)	40 (33%)	
18-49	176 (47%)	68 (47%)	42 (38%)	66 (55%)	
50-64	27 (7.2%)	11 (7.5%)	9 (8.2%)	7 (5.8%)	
≥65	8 (2.1%)	4 (2.7%)	3 (2.7%)	1 (0.8%)	
**Female**	195 (52%)	72 (49%)	55 (50%)	68 (57%)	0.38
**Ethnicity**					0.45
Ladino / Mestizo	335 (89%)	133 (91%)	94 (85%)	108 (90%)	
Indigenous	5 (1.3%)	0 (0%)	2 (1.8%)	3 (2.5%)	
Other/Don’t know	36 (9.6%)	13 (8.9%)	14 (13%)	9 (7.5%)	
**Attends school**	45 (12%)	17 (12%)	13 (12%)	15 (13%)	1
**Works outside of home**	121 (32%)	46 (32%)	32 (29%)	43 (36%)	0.33
Work environment					0.71
Outdoors	101 (83%)	38 (83%)	27 (84%)	36 (84%)	
Indoors	9 (7.4%)	5 (11%)	1 (3.1%)	3 (7.0%)	
Both	11 (9.1%)	3 (6.5%)	4 (13%)	4 (9.3%)	
Occupation					0.91
Field/Farm	78 (64%)	31 (67%)	20 (63%)	27 (63%)	
Planta/Empacadora	22 (18%)	9 (20%)	5 (16%)	8 (19%)	
Other	21 (17%)	6 (13%)	7 (22%)	8 (19%)	
**Pre-existing conditions**^d^	85 (23%)	25 (17%)	27 (25%)	33 (28%)	0.66
Liver disease	3 (0.8%)	0 (0%)	0 (0%)	3 (2.5%)	0.04
**Reported taking medication**	42 (11%)	10 (6.8%)	16 (15%)	16 (13%)	0.85
**COVID-19 vaccine brand**^e^					0.24
Astra Zeneca	23 (13%)	6 (9.1%)	11 (21%)	6 (9.7%)	
Moderna	110 (61%)	41 (62%)	28 (54%)	41 (66%)	
Pfizer	14 (7.8%)	5 (7.6%)	6 (12%)	3 (4.8%)	
Sputnik	4 (2.2%)	0 (0%)	1 (1.9%)	3 (4.8%)	
Heterologous	29 (16%)	14 (21%)	6 (12%)	9 (15%)	
None	196	80	58	58	
**Vaccination status**^f^					0.03
Unvaccinated/no evidence of vaccination	79 (30%)	25 (25%)	17 (23%)	37 (42%)	
Partially vaccinated	55 (21%)	24 (24%)	19 (25%)	12 (13%)	
Fully vaccinated	46 (17%)	17 (17%)	16 (21%)	13 (15%)	
Fully vaccinated + booster(s)	86 (32%)	36 (35%)	23 (31%)	27 (30%)	86 (32%)
Ineligible	110	44	35	31	
**Vaccinated within 6 months of exposure**^g^					0.26
Unvaccinated/last dose ≥6 months	145 (39%)		74 (67%)	71 (59%)	
Any dose <6 months	85 (23%)		36 (33%)	49 (41%)	
**Baseline serology**					<0.01
Negative	51 (14%)	0 (0%)	16 (15%)	35 (29%)	
Positive	160 (43%)	16 (11%)	70 (64%)	74 (62%)	
Not tested/unknown	165 (44%)	130 (89%)	24 (22%)	11 (9.2%)	

a Median (interquartile range); n (%).

b One household with tertiary transmission only ^c^From Wilcoxon rank sum test (medians), chi-square test, or Fisher’s exact test (cell size <5) between uninfected and infected households or household members (household characteristics or individual characteristics) in infected households.

d Asthma, anemia, cardiovascular disease, diabetes, kidney disease, liver disease, neurologic disease, body mass index > 30 kg/m^2^; also tested individually (all *P*-values >0.05, except liver disease).

e Percentages are among those vaccinated to highlight brand differences.

f Percentages here are among those eligible for COVID-19 vaccination in Guatemala at that time (≥12 years old) but the denominator also includes 11 who received vaccinations regardless of eligibility guidelines.

g At time of first household infection for SARS-CoV-2 infected households.

### Secondary attack rate among contacts

**Figure 3:**
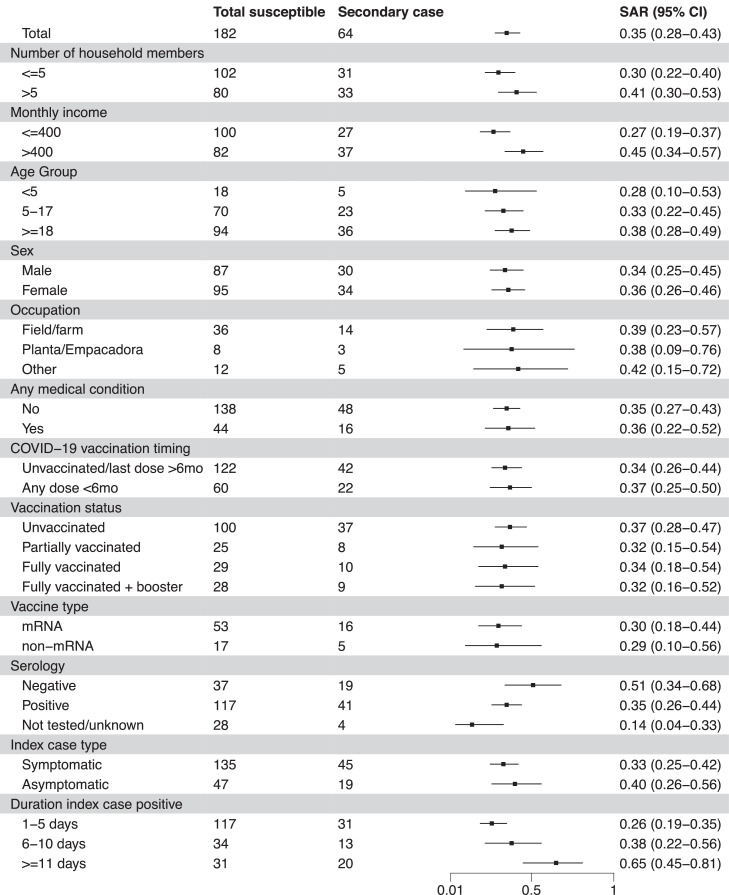
Secondary attack rates for SARS-CoV-2 by characteristics of interest.

CI, confidence interval; SAR, secondary attack rate.

Of 182 household contacts of index infections, 64 (35%) contacts became subsequently infected with SARS-CoV-2; 56 (88%) of these 64 had neither anti-N IgG antibodies (19, 30%) nor COVID-19 vaccination (n = 37, 58%) at baseline. The estimated SAR in the first SARS-CoV-2 household introduction was 0.35 (95% CI 0.28-0.43), the second 0.29 (95% CI 0.17-0.43), and no secondary transmission occurred in the third. SARs by age group and occupation ranged from 0.27-0.65 (Figure 3). In the multivariable regression model, the risk of secondary transmission to household contacts was not increased for contacts of index infections who tested positive for 6-10 days (RR = 1.40, 95% CI 0.77-2.57) but was 2.12 times higher among contacts of index infections positive for ≥11 days (RR = 2.12, 95% CI 1.29-3.49, unadjusted SAR = 0.65, 95% CI 0.45-0.81), when compared to contacts of index infections positive for ≤5 days. No other household or individual-level characteristics were associated with a statistically significant risk of transmission (Table 2)**.**

**Table 2:** Risks of SARS-CoV-2 infection to household contacts.

Household (n = 77) characteristics	Infected households	Infected households with secondary transmission	Unadjusted RR		Adjusted RR	
	N = 48 (62%)^a^	N = 29 (38%)^a^	RR (95% CI)	*P*-value	RR (95% CI)	*P*-value
**Number of household members**	5 (4, 6)	5 (4, 6)	1.01 (0.93-1.11)	0.78		
**Number of adults**	2 (2, 3)	2 (2, 4)	1.08 (0.95-1.23)	0.25		
**Square meters per person**	2 (1, 5)	2 (1, 5)	0.99 (0.96-1.02)	0.41		
**Rooms per person**	0.80 (0.60, 1.18)	0.80 (0.60, 1.00)	0.87 (0.48-1.58)	0.65		
**Beds per person**	0.75 (0.60, 1.00)	0.70 (0.60, 1.00)	0.65 (0.19-2.27)	0.50		
**Windows per person**	0.50 (0.33, 0.83)	0.55 (0.33, 0.83)	1.26 (0.73-2.16)	0.41		
**Monthly income (US $)**	387 (323, 452)	439 (348, 452)	1.00 (1.00-1.00)	0.00		

Susceptible individual (n = 182) characteristics^b^	Susceptible house members	Secondary cases				
	N = 182	N = 64 (35%)				
**Age group**						
<18	88 (48%)	28 (44%)			REF	
≥18	94 (52%)	36 (56%)	1.20 (0.84-1.73)	0.32	1.36 (0.93-1.98)	0.11
**Female**	95 (52%)	34 (53%)	1.04 (0.69-1.55)	0.86	1.08 (0.76-1.55)	0.66
**Attends school**	22 (12%)	8 (13%)	1.04 (0.56-1.92)	0.90		
**Works outside of home**	56 (31%)	22 (34%)	1.18 (0.80-1.75)	0.41		
**Any medical condition(s)**^c^	44 (24%)	16 (25%)	1.05 (0.70-1.56)	0.83		
**COVID-19 vaccination status**						
Unvaccinated	100 (55%)	37 (58%)	REF			
Partially vaccinated	25 (14%)	8 (13%)	0.86 (0.47-1.61)	0.65		
Fully vaccinated	29 (16%)	10 (16%)	0.93 (0.53-1.64)	0.81		
Fully vaccinated + booster	28 (15%)	9 (14%)	0.87 (0.51-1.49)	0.61		
**Vaccination timeliness**						
Unvaccinated/last dose ≥6 months	122 (67%)	42 (66%)	REF		REF	
Any dose <6 months	60 (33%)	22 (34%)	1.07 (0.71-1.59)	0.76	0.85 (0.56-1.30)	0.46
**Baseline serology**^d^						
Negative	37 (20%)	19 (30%)	REF		REF	
Positive	117 (64%)	41 (64%)	0.68 (0.43-1.08)	0.10	0.70 (0.42-1.18)	0.19
Not tested/unknown	28 (15%)	4 (6.3%)	0.28 (0.08-0.91)	0.03	0.35 (0.10-1.18)	0.09
**Index case status**						
Symptomatic	135 (74%)	45 (70%)	REF			
Asymptomatic	47 (26%)	19 (30%)	1.21 (0.74-1.99)	0.45		
**Index case number of days positive detected**						
1-5 days	117 (64%)	31 (48%)	REF		REF	
6-10 days	34 (19%)	13 (20%)	1.44 (0.78-2.67)	0.24	1.40 (0.77-2.57)	0.27
≥11 days	31 (17%)	20 (31%)	2.43 (1.49-3.97)	0.00	2.12 (1.29-3.49)	<0.01
**Frequency of isolation**^e^	0 (0, 0)	0 (0, 0)	0.37 (0.03-5.06)	0.46		
**Frequency physical contact**^e^	0.80 (0.0, 1.0)	1.0 (0.0, 1.0)	1.04 (0.52-2.09)	0.91		
**Frequency mask use**^e^	0 (0, 0)	0 (0, 0)	0 (0-0)	0.00		

CI, confidence interval; RR, risk ratio.

a Median (IQR); n (%).

b Susceptible household member characteristics unless otherwise noted.

c Included participants with at least one of the following conditions reported: asthma, anemia, cardiovascular disease, diabetes, kidney disease, liver disease, neurologic disease, obesity, pulmonary disease.

d Anti-nucleocapsid immunoglobulin G.

e Percentage of times the activity was reported within 10 days of household infection.

## Discussion

We found high SARS-CoV-2 attack rates among agricultural worker households in Guatemala. Almost three-quarters of index cases (71%) were adults—56% of those working outside of the home, and over a third of those adults were enrolled in our separate agricultural worker (AGRI) cohort. More than half of households (58%) had at least one SARS-CoV-2 infection and nearly two-thirds (63%) of these households had secondary transmission during 6 months of follow up with two out of five households having had reintroductions of SARS-CoV-2. More than half of infected individuals developed symptoms. Such attack rates can demonstrate the vulnerability to SARS-CoV-2 infection of agricultural workers and their households as they worked to sustain food supply during the COVID-19 pandemic.

The SARs we report for these agricultural communities are comparable to other SARs reported for Delta and Omicron variants [23,24], but higher than estimates published earlier in the COVID-19 pandemic [11,25]. However, the scarcity of targeted research on SARS-CoV-2 transmission among agricultural workers, aside from our study, hinders the identification of what may be important differences in SARs for this demographic compared to other groups. The unique vulnerabilities of agricultural workers may lead to more severe COVID-19 epidemics (or pandemics), as evidenced by the experiences of Guatemalan agricultural workers [1], who make up 27% of the total Guatemalan workforce of whom 34% are employed informally [26], i.e., without a written contract or formal agreement. Some of these unique vulnerabilities pertain to chronic comorbidities more common in the agricultural sector that may increase disease severity, such as chronic kidney disease [8,27]; other vulnerabilities may increase the risk of infection such as crowded transportation (buses) or work environments (processing plants) as well as economic incentives (pay based on productivity) to under-report symptoms to the employer, and type of occupation within the farm [1].

In our cohort, household contacts were more likely to get sick when the index cases shed SARS-CoV-2 RNA for longer periods. While previous studies have not found associations between duration of shedding and infectiousness [28] the risk of secondary transmission in our cohort more than doubled if SARS-CoV-2 RNA detection continued for longer than 10 days. Previous publications suggest that recovery of replication-competent SARS-CoV-2 after 10 days is rare [29,30]. Nevertheless, we might have identified associations between the duration of shedding SARS-CoV-2 RNA and secondary attack rates if index cases with prolonged viremia had higher initial viral loads or undiagnosed conditions which might have had altered magnitude and length of viral shedding [31,32].

Consistent with other studies [11,12] we observed notable pre-symptomatic or asymptomatic transmission (27%) within our households, indicating that individuals who do not show symptoms likely contribute to the spread of SARS-CoV-2, including through saliva. Previous studies have demonstrated saliva as a reliable specimen for SARS-CoV-2 detection [33]. Saliva from asymptomatic and presymptomatic individuals seems to contribute to transmission; viable virus may be cleared from the nasopharynx but might persist in saliva [34]. Such findings suggest that households of essential workers should not only rely on reactive infection control practices, such as mask-wearing when a household member becomes sick, but should also seek COVID-19 vaccination [35] and maintain good handwashing, surface hygiene, and respiratory hygiene practices when possible. During household infection with symptomatic household members, isolation measures may still be applicable; in Guatemala, it was recommended that symptomatic or infected individuals isolate in a separate room, or, if not possible to maintain a distance of at least 1-2 m from other household members [36]. We were underpowered to demonstrate if COVID-19 vaccination might have decreased the risk of secondary transmission in the population studied. First, most vaccinated individuals in the study received their last COVID-19 vaccine dose more than 6 months before household infection, when the modest effectiveness against infection is known to wane [37]. Second, participants were vaccinated with first-generation vaccines which were less effective against infection with the Omicron and subsequent SARS-CoV-2 variants [38], [39] predominant in Central America during the study period. Third, younger people (<12 years old) in our study were not eligible for COVID-19 vaccination per Guatemalan Ministry of Health guidelines. Last, a substantial proportion of participants with serologic testing exhibited baseline positive anti-N IgG results for SARS-CoV-2, which may have further confounded the protective effect of vaccination in our study [40].

Our study had strengths including the timely, in-person surveys of households when symptoms were reported, high household retention and replacement, weekly saliva sampling irrespective of the presence of symptoms that likely increased asymptomatic and pre-symptomatic case detection (compared to other studies), timely surveying and testing of household infections prompted by symptomatic individuals, accurate COVID-19 vaccination records, and a team of experienced, well-trained field and laboratory staff in this rural community. Our study also had limitations. First, positive test results from asymptomatic index cases would only trigger household contact surveillance after an index case; this would have introduced a couple of days delay in household surveillance while the index case’s sample was being tested for SARS-CoV-2. Second, many household members had previous SARS-CoV-2 infections by September 2021, when we initiated the study. Third, we could not distinguish workplace versus community contagion among cases. Last, we were underpowered to assess SAR by participant age group, sex, and preexisting conditions.

In our study of Guatemalan agricultural workers and their households, we found high attack rates for SARS-CoV-2 with a high proportion of asymptomatic or pre-symptomatic index cases. Longer duration of SARS-CoV-2 RNA shedding correlated with increased risks of secondary SARS-CoV-2 infections. This study in Guatemala suggests that targeting non-pharmaceutical interventions and vaccination campaigns to essential workers in sectors like agriculture could help mitigate the impact of epidemics and pandemics. Advising the community about respiratory hygiene and mask-wearing during epidemics might modestly reduce household respiratory virus transmission [41] (e.g., through saliva). Programs that mitigate the risks of respiratory illness exacerbated by chronic comorbidities, crowded environments, and economic pressures should be considered. Furthermore, public health officials may implement field-based real-time surveillance coupled with rapid diagnostics, as demonstrated here, to monitor these communities for new cases and allow for more targeted messaging and resource allocation regarding preventive measures. Our findings reaffirm that agricultural workers are at high risk of illness during epidemics or pandemics. Given their critical role in food supply chains for global food security, these risks should be monitored with particular care; agricultural workers and their communities should be a vital consideration for pandemic preparedness.

## Declarations of competing interest

The authors declare no conflicts of interest. D.O. has research support from Roche Diagnostics, Merck, and Sanofi Pasteur; the study field team received educational support from Roche Diagnostics.

## Appendix Supplementary materials

Supplementary Figure 1. Weekly SARS-CoV-2 household episodes (n = 64) Image, application 1

## Footnotes

Supplementary material associated with this article can be found, in the online version, at doi:10.1016/j.ijregi.2025.100676.
