# Supplementary figures and images for: SARS-CoV-2 secondary attack rates and risks for transmission among agricultural workers and their households in Guatemala, 2022-2023
Source: IJID Reg. 2025 May 27;16:100676. doi: 10.1016/j.ijregi.2025.100676 (PMC12210297; doi:10.1016/j.ijregi.2025.100676)

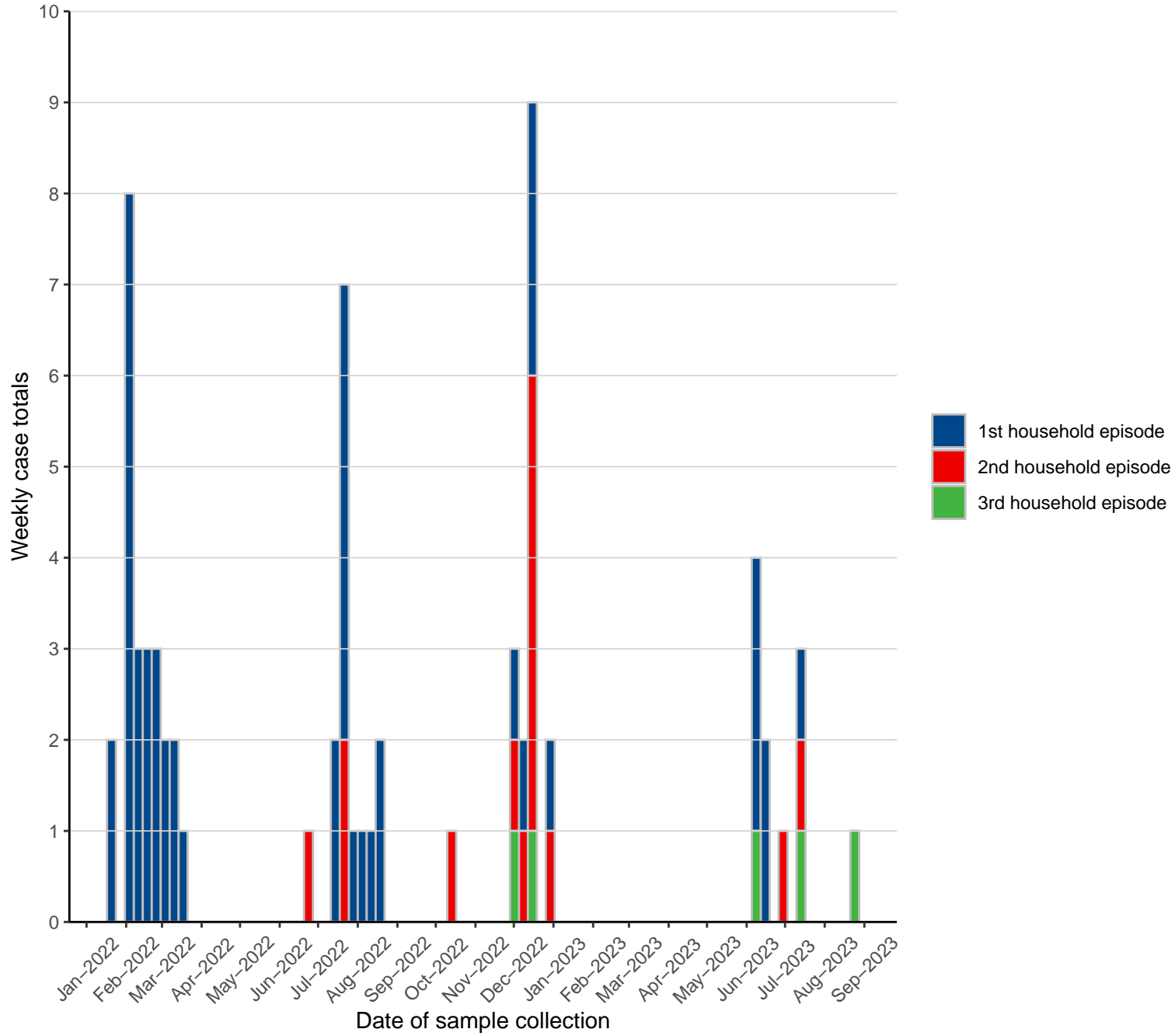

Supplement: Supplementary file 1 — Supplementary Figure 1. Weekly SARS-CoV-2 household episodes (n = 64) [file mmc1.pdf]
